# Clinical Impact of the Time in Therapeutic Range on Early Hospital Readmission in Patients with Acute Heart Failure Treated with Oral Anticoagulation in Internal Medicine

**DOI:** 10.3390/medicina57040365

**Published:** 2021-04-09

**Authors:** Rubén Ángel Martín-Sánchez, Noel Lorenzo-Villalba, Alberto Elpidio Calvo-Elías, Ester Emilia Dubón-Peralta, Cynthia Elisa Chocrón-Benbunan, Carmen María Cano-de Luque, Lidia López-García, María Rivas-Molinero, Cristina Outón-González, Javier Marco-Martínez, Elpidio Calvo-Manuel, Emmanuel Andres, Manuel Méndez-Bailón

**Affiliations:** 1 Internal Medicine Department, Hospital Universitario Clínico San Carlos, Departamento de Medicina Universidad Complutense, Insituto de Investigacion Hospital Clinico San Carlos, 28040 Madrid, Spain; rbnmattins@hotmail.com (R.Á.M.-S.); calvoelias@gmail.com (A.E.C.-E.); esterdubon@gmail.com (E.E.D.-P.); cchocronb@gmail.com (C.E.C.-B.); carmencano93@gmail.com (C.M.C.-d.L.); lidial02@ucm.es (L.L.-G.); rivasmolinero@gmail.com (M.R.-M.); crisouton@gmail.com (C.O.-G.); javiermarco.z@gmail.com (J.M.-M.); ecalvo@ucm.es (E.C.-M.); manuelmenba@hotmail.com (M.M.-B.); 2 Service de Médecine Interne, Diabète et Maladies Métaboliques, Hôpitaux Universitaires de Strasbourg, 1 Place de l’Hôpital, 67000 Strasbourg, France; emmanuel.andres@chru-strasbourg.fr

**Keywords:** heart failure, non-valvular atrial fibrillation, acenocoumarol, time in therapeutic range, readmission

## Abstract

*Background and objectives:* Patients with heart failure (HF) often present with non-valvular atrial fibrillation and require oral anticoagulation with coumarin anticoagulants such as acenocoumarol. The objective of this study was to evaluate the relationship between time in therapeutic range (TTR) and the risk of early readmission. *Materials and Methods:* A retrospective descriptive study was carried out on hospitalized patients with a diagnosis of HF between 2014 and 2018 who had adverse effects due to oral anticoagulation with acenocoumarol (underdosing, overdosing, or hemorrhage). Clinical, analytical, therapeutic, and prognostic variables were collected. TTR is defined as the duration of time in which the patient’s International Normalized Ratio (INR) values were within a desired range. Early readmission was defined as readmission within 30 days after hospital discharge. Patients were divided into two groups depending on whether or not they had a TTR less than 60% (TTR < 60%) over the 6 months prior to the adverse event. *Results:* In the cohort of 304 patients, the mean age was 82 years, 59.9% of the patients were female, and 54.6% had a TTR < 60%. Patients with TTR < 60% had a higher HAS-BLED score (4.04 vs. 2.59; *p* < 0.001) and INR (6 vs. 5.31; *p* < 0.05) but lower hemoglobin (11.67 vs. 12.22 g/dL; *p* < 0.05). TTR < 60% was associated with early readmission after multivariate analysis (OR: 2.05 (CI 95%: 1.16–3.61)). They also had a higher percentage of hemorrhagic events and in-hospital mortality but without reaching statistical significance. *Conclusions:* Patients with HF and adverse events due to acenocoumarol often have poor INR control, which is independently associated with a higher risk of early readmission.

## 1. Introduction

The incidence of heart failure (HF) is progressively increasing in daily clinical practice and is often associated with non-valvular atrial fibrillation [1] (NVAF), requiring anticoagulant treatment [2]. Vitamin K antagonists (VKAs) have been used for many years as the cornerstone of stroke prevention in non-valvular atrial fibrillation (NVAF). However, treatment with VKAs is associated with a narrow therapeutic range that requires frequent monitoring of coagulation parameters, drug and food interactions, and a significant risk of bleeding, including intracranial haemorrhage (ICH).

Direct-acting oral anticoagulants (DOACs) have emerged as therapeutic alternatives in NVAF as they overcome many of the inherent disadvantages of VKAs. Based on the efficacy, safety, and convenient administration of DOACs, the current international guidelines recommend these agents as preferable to VKAs for most patients with NVAF for whom oral anticoagulation (OAC) is indicated [2]. Despite this, the use of VKAs is still significantly more predominant than DOACs in Spain [3,4] even if it has been reported that approximately 40% of AF patients on VKA treatment have poor control of anticoagulation [5,6,7]. This situation could result from the fact that the prescription of direct-acting anticoagulants in atrial fibrillation in Spain is subject to state funding through a pharmaceutical regulation. Not all patients wish to change treatment and assume its cost.

In this context, Acenocoumarol is currently the most frequently prescribed anticoagulant agent in Spain. Acenocoumarol should be periodically monitored using the International Normalized Ratio (INR), and the adequacy of anticoagulant therapy can be described using the time in therapeutic range (TTR). TTR can be calculated by the Rosendaal method or by the direct method, and a value below 65% or 60%, respectively, after 6 months of treatment would indicate a suboptimal control of the INR [8].

One of the disadvantages of the treatment with acenocoumarol is the difficulty of maintaining an adequate TTR. There are several studies reporting the evaluation of the control of anticoagulation in patients with NVAF treated with acenocoumarol. One study found that 40% of the patients treated in primary care facilities showed inadequate anticoagulation control in the previous 12 months [9], while another study noted that despite the high thromboembolic risk, only 47% of the patients treated in Internal Medicine and Neurology consultations were well controlled [10]. In addition, in a systematic review of the literature, Vestergaard et al. concluded that higher TTR values were associated with a lower percentage of complications, especially hemorrhages [11].

HF leads to a deterioration of the patient’s quality of life as well as an increased risk of complications and hospital admissions, despite improvements in management and pharmacotherapy [12,13]. Anticoagulation with anti-vitamin K drugs in these patients could worsen this situation by increasing the risk of hospitalization in those who have suboptimal anticoagulation control [14].

The main objective of our study was to define the clinical characteristics of patients with non-atrial valvular fibrillation and heart failure who presented an adverse reaction to oral anticoagulation with acenocumarol. The second was to evaluate the relationship between TTR and early hospital readmission (defined as admission within thirty days after hospital discharge) as well as the relationship between TTR and the risk of mortality and adverse events due to anticoagulation.

## 2. Methods

We conducted an observational and retrospective cohort study including the data from patients admitted to San Carlos University Hospital between January 2014 and August 2018. We accessed the Spanish Minimum Basic Data Set (CMBD) [15], which is a database established in 1987 of characteristics of patients admitted to nearly 300 hospitals in Spain that contains information including demographics such as age, sex, and residence, episodes of hospitalization, diagnoses, and procedures performed during hospitalization coded by either ICD9CM or ICD10 systems. We included patients with main diagnoses of the CMBD CI (I428) and Adverse effect of anticoagulation (T24.515) according to ICD-9 coding from January 2014 to December 2015, and congestive IC (I50) and Adverse effect of anticoagulants (T45.515) according to ICD-10 coding from January 2016 to August 2018. Patients with heart failure and who have presented an adverse reaction to anticoagulation (bleeding, overdose) as well as those with the diagnosis of NVAF and on treatment with acenocoumarol for more than six months before the adverse event were included. To avoid duplicating patients, we checked the medical history numbers. If two history numbers coincided in our database, we included also the first hospitalization. In these cases, the duplicate history number always corresponded to the readmission of the same patient.

Clinical variables encompassing the CHA2DS2-VASc and HAS-BLED scales were collected [16,17]. This included the age, history of HF, arterial hypertension, diabetes mellitus, stroke and vascular ischemia, abnormal renal function (serum creatinine greater than 200 μmol/L, history of renal transplantation, or dialysis treatment), abnormal liver function (bilirubin two times above the normal range, aspartate transaminase, aspartate transaminase, alkaline phosphatase three times above the normal range, or the presence of cirrhosis), anemia or previous bleeding episodes, labile INR defined as the TTR calculated by the direct method less than 60% in the last 6 months prior to the adverse event, and a previous history of alcohol consumption or use of non-steroidal anti-inflammatory or antiplatelet agents.

The analytical variables collected were the INR at the time of the adverse event, hemoglobin, and the glomerular filtration rate according to the CKD-EPI formula. Diagnostic variables were considered as overdosing (if the INR value was greater than 3), underdosing (if the INR value was less than 2), and hemorrhage. Hemorrhage was classified as cerebral, gastrointestinal, or any other type (encompassing other bleeding complications). Therapeutic variables were collected and classified into two groups depending on whether the patient’s therapeutic management was performed during admission (temporary suspension of anticoagulation, administration of vitamin K, or transfusion of red blood cells) or discharge (permanent anticoagulation discontinuation, maintenance of acenocoumarol, switch to heparin or direct action anticoagulants, and continuation of antiplatelet therapy). The average hospital stay, early readmission, and in-hospital mortality were also analyzed.

The study population was subsequently divided into two groups depending on whether or not the TTR was less than 60% (TTR < 60%).Variables were compared and analyzed between both groups. Quantitative variables are presented as mean and standard deviation (SD) and qualitative variables as counts and percentages. Student T-test was used for independent samples of quantitative variables and the chi-square test was used for comparison of qualitative variables. A bivariate analysis was performed using clinical variables in relation to anticoagulation (age, sex, CHADs Vasc, HAS-BLED, glomerular filtration rate, and TTR) in readmitted and non-readmitted patients. Those variables showing a statistical significance in this model were subsequently entered in a multivariate analysis.

A multivariate analysis by logistic regression was performed with the variable “readmission” as a dependent variable and the covariates that were either statistically significant in the bivariate analysis or had a prognostic relevance such as age, TTR < 60%, and glomerular filtration rate. Statistical significance was set at *p* < 0.05. Statistical analysis was performed using SPSS (IBM Corporation. Released 2016, IBM SPSS Statistics for Windows, v24.0, IBM Corporation, Armonk, NY, USA). The study was approved by the Clinical Research Ethics Committee of the hospital. The data collected were treated in accordance with the Organic Law IS/2016 for the protection of personal data.

## 3. Results

A total of 357 patients were initially enrolled, but only 304 met the inclusion criteria. The mean age was 82.4 ± 7.9 years, and females represented 59.9% of the sample. Mean CHA2DS2-VASc score was 4.7 ± 1.3 and mean HAS-BLED scale was 3.37 ± 1.44. The mean values of analytical values were: INR at the time of the adverse event 5.69 ± 2.82; hemoglobin 11.98 ± 1.95 g/dL, and glomerular filtration rate (CKD-EPI) 49.67 ± 21.51 mL/min.

In evaluation of diagnostic variables, 2.57% of patients were noted to have underdosing of acenocoumarol (mean INR value 1.41 ± 0.2) compared to 97.43% of patients with acenocoumarol overdosing (mean INR value 5.73 ± 3.27). In addition, 11.2% (*n* = 34) of patients presented any kind of bleeding complication: one intracranial hemorrhage, 13 gastrointestinal bleeding, and 20 other types of hemorrhage. In the sample, 23.16% of the patients were readmitted within 30 days after hospital discharge, and in-hospital mortality was 7.38%. The leading cause of early readmission in the patients in our study was heart failure, in 45 of 72 of the patients who were readmitted, followed by bleeding from any cause, in 16 of 72 of the patients, and other causes, in 11 of 72 patients. (Table 1).

Table 2 shows the results from the bivariate analysis based on a TTR < 60% or not. The group with TTR value < 60% represented 54.6% (*n* = 166) of the patients and the group with a TTR value greater than 60% represented 45.4% (*n* = 138) of the patients. A higher HAS-BLED scale score (4.04 ± 1.35 vs. 2.59 ± 1.09; *p* < 0.001) was found in patients with a TTR < 60% compared to patients with TTR > 60%. They also showed higher INR levels at the time of the event (6 ± 2.82 vs. 5.31 ± 2.53; *p* < 0.05) and lower hemoglobin levels (11.67 ± 1.96 g/dL vs. 12.22 ± 1.88 g/dL; *p* < 0.05). In patients with a TTR value < 60%, more cases of overdosing (52.3% vs. 42.8%; *p* = 0.286) and hemorrhagic events (7.2% vs. 4.9%; *p* = 0.527) were noted, although this did not reach statistical significance. Regarding management at discharge, anticoagulant treatment with acenocoumarol was continued in fewer patients with a TTR <60% compared to those with a TTR > 60% (66.8% vs. 79.8%; *p* < 0.05), although the treatment was more frequently switched to another type of anticoagulant drug (heparin or direct-acting oral anticoagulants) (18.9% vs. 10.5%; *p* < 0.05). The switch to heparin (13.2% vs. 7.9%; *p* < 0.05) or direct-acting anticoagulants (7.2% vs. 2.9%; *p* < 0.05) was relevant in the first group. Early hospital readmission was higher in patients with a TTR < 60% (16.1% vs. 7.6%; *p* < 0.05), and a similar trend in in-hospital mortality (5.6% vs. 2.6%; *p* = 0.16) was observed.

In the bivariate analysis, age, glomerular filtration rate and the TTR < 60% were the variables associated to readmission in patients with heart failure, NVAF, and adverse reaction to oral anticoagulation (*p* = 0.04, *p* = 0.01, and *p* = 0.40 respectevely (Table 3). These results were also confirmed in the multivariate analysis (Table 4).

## 4. Discussion

In our study, 54.6% of the patients had a TTR < 60%, similar to that reported in the ALADIN study. However, this result was higher than that of the ANFAGAL study [18], in which 41.5% of the patients had a TTR < 60%. The importance of improving control during anticoagulation therapy was also shown in another study showing a mean TTR of 40% with a mean anticoagulation treatment of 5.4 years in anticoagulant treatment [19].

An important factor leading to this result in the current study was the mean age of the population studied (82 years). Anticoagulation in elderly patients represents a significant challenge because of the frequent association with other medical conditions that can modify not only the therapy indication but also the type and dosage of drug, tolerance, compliance, safety profile, and the expected results. Among these health determinants, it is important to note the interplay of frailty, disability, comorbidity, polypharmacy, cognitive impairment, risk of falling, nursing home residence, nutritional status, oral feeding problems, sensory disorders, and personal and social issues.

The association between the diagnosis of acute HF and a lower value of TTR has been reported previously [20]. Kose et al. described a relationship between the fluctuation of atrial natriuretic peptide levels in patients with HF with poor control of INR, emphasizing that these patients would benefit from closer monitoring [21]. However, there are other factors that influence the quality of anticoagulation. Faricloth et al., in a retrospective study evaluating the TTR within the prior 19 months, found that diet, non-compliance, and drug interactions could be precipitating factors [22]. Eggebrecht et al. also reported that patients with co-morbidities and polypharmacy had increased numbers of adverse events when treated with anti-vitamin K therapy [23].

Risk–benefit is considered before switching from VKAs to DOACs in patients with poor INR control in our country [24]. Despite this, the results of our study showed that more than half of the patients with TTR < 60% are maintained on acenocoumarol treatment. A similar result was reported in another study conducted in hospitalized patients receiving anticoagulant therapy with acenocoumarol, in which INR control also worsened during hospitalization and was associated with a longer hospital stay [25]. Anemia, bleeding, and a higher number of hospitalizations was associated with warfarin treatment discontinuation in patients with NVAF [26].

The percentage of bleeding events in our study was low (12.2%) probably as a result of the sample’s size. Patients with a TTR < 60% were more prone to develop a hemorrhagic event. Inoue et al. had previously described this relationship in a study with elderly patients with NVAF in which TTR below 40% increased the risk of bleeding, while in patients with TTR > 70%, the thromboembolic risk was reduced [27]. Similarly, Senoo and Lip demonstrated a negative correlation between TTR and any clinically relevant hemorrhagic event [28]. In a retrospective study of 53,935 patients in Finland, a lower TTR was associated with an increased risk of intracranial hemorrhage [29]. However, there are no studies evaluating this relationship in patients with HF.

The mortality rate in our cohort was higher in patients with a TTR < 60% without reaching statistical significance probably due to the small simple size. In a recent Spanish study, INR in the suboptimal range at admission in patients with HF treated with acenocoumarol for NVAF was associated with a higher risk of mortality in long-term follow-up [30]. An American retrospective study showed the relationship between different causes of mortality (hemorrhages, strokes) and poor warfarin treatment control [31]. Similarly, Rivera-Caravaca observed in his study an increase in the risk of mortality and significant clinical events (major bleeding, systemic embolism, HF) in those patients with poor INR control [32].

The TTR < 60% was significantly associated in the multivariate analysis with early readmission. There are few studies analyzing this parameter in patients with HF and treated with anti-vitamin K anticoagulants. As previously mentioned, Rivera-Caravaca et al. demonstrated that alterations in TTR were associated with a higher number of clinical events such as HF. Bhattarai et al. evaluated the impact of warfarin and direct-acting anticoagulants on hospital readmission within 30 days after hospital discharge and found that readmissions rates were higher in patients treated with warfarin [33]. Mahesh et al. analyzed readmissions after coronary angiography and observed a higher percentage of readmission rates in those treated with warfarin at discharge [34]. Brunetti et al. demonstrated the importance of patient participation in education programs for the management of anticoagulants such as warfarin in order to reduce hospital readmissions at 90 days [35].

The present study has some limitations due to its design. First, the sample size is small to assess bleeding events and mortality. Establishing causality–effect relationships between the quality of anticoagulation and the risk of early readmission is complicated, so these results should be carefully interpreted. In addition, the diagnosis of HF was obtained from the CMBD diagnostic coding system, without taking into account the proBNP value and transthoracic echocardiogram report. Of note, there is no control group of patients with HF on treatment with acenocoumarol without adverse events. Even though this is a retrospective study, the clinical variables of our administrative base were reviewed by physicians who assigned the outcome variable of both readmission and mortality, which may add an additional clinical value

In accordance with the latest clinical practice guidelines for the treatment of heart failure, our study reinforces the need to switch from antivitamin K drugs to new oral anticoagulants. In the case of not being able to do so due to lack of funding for these drugs, we recommend stricter anticoagulation control in patients with TTR < 60%.

## 5. Conclusions

Although previous studies have evaluated the impact between TTR and anticoagulation complications in patients with HF, our study is the first to specifically analyze the relationship between TTR and the risk of readmission within 30 days after hospital discharge. In those patients admitted with HF who had an adverse event due to anticoagulation with acenocoumarol, a TTR < 60% was independently associated with the risk of early readmission. A higher percentage of in-hospital mortality and hemorrhagic complications was also observed in these patients, although statistical significance was not reached. More than half of the patients with TTR < 60% continued acenocoumarol at hospital discharge. Improved control of anticoagulation therapy could reduce these complications.

## Figures and Tables

**Table 1:** Clinical characteristic in patients with heart failure (HF) and adverse reaction toanticogulation.

Variable	
Age (years) (mean, SD)	82.48 ± 7.90
Female (*n*, %)	181 (59.5%)
Medical antecedents	
Arterial hypertension (*n*, %)	269 (88.5%)
Diabetes mellitus (*n*, %)	132 (43.4%)
Heart failure (*n*, %)	272 (89.5%)
Previous stroke or transient vascular accident (*n*, %)	46 (15.1%)
Previous vascular ischemia (*n*, %)	78 (25.6%)
Abnormal renal function (*n*, %)	27 (8.9)
Abnormal liver function (*n*, %)	9 (3%)
Anemia/hemorrhage (*n*, %)	104 (34.2%)
INR lability (TTR < 60%)(*n*, %)	166 (54.6%)
INR at the time of the event (mean, SD)	5.69 ± 2.82
CHA2DS2-VASc (mean, SD)	4.70 ± 1.30
HAS-BLED (mean, SD)	3.37 ± 1.44
Hemoglobin at admission (mean, SD) (g/dL)	11.98 ± 1.95
Glomerular filtration rate at admission (CKD-EPI, mL/min/1.73m^2^) (mean, SD)	49.67 ± 21.51
Anti-platelet treatment (*n*, %)	46 (15.1%)
Acenocoumarol underdose (*n*, %)	9 (0.03%)
Acenocoumarol overdose (*n*, %)	289 (95%)
Intracranial hemorrhage (*n*, %)	1 (0.3%)
Gastrointestinal bleeding (*n*, %)	13 (4.3%)
Other hemorrhages (*n*, %)	20 (6.6%)
Total hemorrhage(*n*, %)	37 (12.2%)
Discontinuation of anticoagulation during hospitalization (*n*, %)	227 (74.7%)
Vitamin K (*n*, %)	49 (16.1%)
Blood transfusion (*n*, %)	25 (8.2%)
Anticoagulation discontinued at discharge (*n*, %)	12 (3.9%)
Anticoagulation maintained at discharge (*n*, %)	243 (79.9%)
Anticoagulation modification at discharge (*n*, %)	22 (7.2%)
Switch to heparin at discharge (*n*, %)	33 (10.8%)
Switch to direct anticoagulant at discharge (*n*, %)	16 (5.3%)
Anti-platelet at discharge (*n*, %)	35 (11.5%)
Mean length of stay (days) (mean, SD)	9.13 ± 8.7
Early readmission (*n*, %)	72 (23.7)
In hospital mortality (*n*, %)	25 (8.2%)

**Table 2:** Bivariate analysis of patients with HF, adverse reaction to anticoagulation, and therapeutic range (TTR) higher or lower than 60%.

Variables	TTR < 60% *n* = 164	TTR > 60% *n* = 140	*p*
Age (mean, SD)	83.45 ± 7.88	83.38 ± 7.98	0.95
Female (*n*, %)	98 (32.2%)	83 (27.3%)	0.08
CHADSVASC (mean, SD)	4.79 ± 1.35	4.63 ± 1.23	0.21
HAS-BLED (mean, SD)	4.04 ± 1.35	2.59 ± 1.11	<0.05
Labile INR (TTR < 60%) (mean, SD)	6.00 ± 2.71	5.31 ± 2.92	<0.05
Glomerular filtration rate at admission (CKD-EPI, mL/min/1.73 m^2^) (mean, SD)	48.98 ± 22.00	0.49 ± 20.98	0.54
Hemoglobin at admission (g/dL) (mean, SD)	11.67 ± 1.95	12.22 ± 1.91	<0.05
Acenocoumarol underdose (*n*, %)	3 (0.9%)	6 (1.9%)	0.45
Acenocoumarol overdose (*n*, %)	161 (52%)	128 (42%)	0.14
Hemorrhage (*n*, %)	21 (7.5%)	15 (5%)	0.32
Anticoagulation discontinued at admission (*n*, %)	71 (23.3%)	44 (14.4%)	0.06
Vitamin K (*n*, %)	32 (10.5%)	17 (5.6%)	0.1
Blood transfusion (*n*, %)	15 (4.9%)	10 (3.3%)	0.57
Anticoagulation discontinued at discharge (*n*, %)	12 (3.9%)	7 (2.3%)	0.26
Anticoagulation maintained at discharge (*n*, %)	110 (36.1%)	112 (36.8%)	<0.05
Anticoagulation modified at discharge (*n*, %)	31 (10.1%)	15 (4.9%)	<0.05
Switch to heparin at discharge (*n*, %)	22 (7.2%)	11 (3.6%)	<0.05
Switch to direct anticoagulant at discharge (*n*, %)	12 (3.9%)	4 (1.3%)	<0.05
Early readmission (*n*, %)	49 (16.1%)	23 (7.6%)	<0.05
In hospital mortality (*n*, %)	17 (5.6%)	8 (2.6%)	0.1

**Table 3:** Bivariate analysis between HF and non-valvular atrial fibrillation (NVAF) patients with and without hospital readmission within 30 days and adverse reaction to oral anticoagulation.

Variables	No Readmission *n* = 232	Readmission *n* = 72	*p*
Age (mean, SD)	83.00 ± 7.5	80.83 ± 8.78	0.040
Female (*n*, %)	143 (61.6%)	38 (58.8%)	0.218
CHADSVASC (mean, SD)	4.65 ± 1.22	4.74 ± 1.52	0.085
HAS-BLED (mean, SD)	3.33 ± 1.41	3.56 ± 1.53	0.22
TTR < 60% (*n*, %)	117 (54.4%)	49 (68.1%)	0.010
Glomerular filtration rate at admission (CKD-EPI, mL/min/1.73 m^2^) (mean, SD)	51.69 ± 21.49	42.73 ± 21,16	0.041
Hemoglobin at admission (g/dL) (mean, SD)	11.99 ± 1.91	12.01 ± 2.03	0.93

**Table 4:** Multivariate analysis of factors associated with early rehospitalization in patients with HF and adverse reaction to anticoagulation.

Variables	ODDS Ratio	95%CI	*p*
TTR < 60%	2.05	1.16–3.61	0.013
Age	1.03	1.00–1.07	0.029
Glomerular filtration rate	1.01	1.00–1.02	0.024
